# Comparison of acid-fast stain and culture for *Mycobacterium tuberculosis* in pre- and post-bronchoscopy sputum and bronchoalveolar lavage in HIV-infected patients with atypical chest X-ray in Ethiopia

**DOI:** 10.4103/1817-1737.36549

**Published:** 2007

**Authors:** Getachew Aderaye, Haimanot G/Egziabher, Abraham Aseffa, Alemayehu Worku, Lars Lindquist

**Affiliations:** Department of Internal Medicine, Medical Faculty, Addis Ababa University, Ethiopia; and Department of Medicine, Division of Infectious Diseases, Kaolinska Institute, Sweden; * *Armauer Hansen Research Institute, Addis Ababa, Ethiopia*; ** *Department of Community Health, Addis Ababa, University, Ethiopia*; *** *Department of Medicine, Division of Infectious Diseases, Karolinska Institute at Karolinska University Hospital, Sweden*

**Keywords:** Acid-fast stain, bronchoalveolar lavage, concentration technique, *M. tuberculosis*, post-bronchoscopy, pre-bronchoscopy

## Abstract

**BACKGROUND:**

Smear-negative tuberculosis occurs more frequently in human immunodeficiency virus (HIV)-infected patients than in non-HIV-infected patients. Besides, there are substantial numbers of patients who cannot produce sputum, making the diagnosis of pulmonary tuberculosis (PTB) difficult.

**AIMS:**

To evaluate the relative yield of pre- and post-bronchoscopy sputum and bronchoalveolar lavage (BAL) in ‘sputum smear’-negative, HIV-positive patients.

**SETTINGS:**

A tertiary care referral hospital in Addis Ababa.

**MATERIALS AND METHODS:**

Acid-fast stain (AFS) using the concentration technique was done on 85 pre-bronchoscopy sputum and 120 BAL samples. Direct AFS was done on all BAL and 117 post-bronchoscopy sputum samples. Culture for *Mycobacterium tuberculosis* (MTB) was done for all sputa and BAL.

**RESULTS:**

MTB was isolated from 26 (21.7%), 23 (19.7%) and 13 (15.3%) of BAL, post- and pre-bronchoscopy sputum cultures respectively. AFS on pre-bronchoscopy sputum using concentration technique and direct AFS on BAL together detected 11 (41%) of the 27 culture-positive cases. In patients who could produce sputum, the sensitivity of pre-bronchoscopy sputum culture (13/85, 15.3%) was comparable to BAL (12/85, 14%) and post-bronchoscopy sputum (12/85, 14%). In patients who could not produce sputum, however, both BAL (12/35, 40%) and post-bronchoscopy sputum (12/32, 31.4%) detected significantly more patients than those who could produce sputum (*P*=0.002, *P*=0.028 respectively).

**CONCLUSION:**

In HIV-infected patients, AFS by concentration method on pre-bronchoscopy sputum and direct AFS on BAL in patients who cannot produce sputum are the preferred methods of making a rapid diagnosis. BAL culture seems to add little value in patients who can produce sputum; therefore, bronchoscopy should be deferred under such circumstances.

The diagnosis of tuberculosis continues to be a challenge in developing countries. The human immunodeficiency syndrome (HIV) has worsened the situation by decreasing the sensitivity of sputum smear for acid-fast bacillus (AFB). As a result, the prevalence of smear-negative tuberculosis is on the rise.[1–3] Besides, both chest radiography and constitutional symptoms of tuberculosis have lost their relative diagnostic value because of atypical presentation and shared symptoms with other pulmonary opportunistic infections.[4,5] In patients who can produce sputum, several studies have demonstrated that sputum acid-fast stain (AFS) after concentration with sodium hypochlorite partially restores the already lost sensitivity of sputum direct microscopy.[6,7] However, many patients with suspected pulmonary tuberculosis (PTB) do not produce sputum spontaneously, making the diagnosis difficult. The current study was undertaken to evaluate the relative yield of pre-bronchoscopy sputum for AFB after concentration with a bleaching agent, pre- and post-bronchoscopy sputum culture and bronchoalveolar lavage (BAL) smear and culture for the diagnosis of *M. tuberculosis* in sputum AFB smear-negative, HIV-positive Ethiopian patients with atypical chest X-ray.

## Materials and Methods

The study was conducted at Tikur Anbessa University Hospital, a tertiary care referral center in Addis Ababa, Ethiopia. Between March 2005 and July 2006, consecutive HIV-infected patients with atypical chest X-rays and whose sputum smear was negative for AFB or could not produce sputum at all were enrolled into the study. Patients were initially excluded from the study if their chest X-rays were judged as typical for tuberculosis or segmental/ lobar pneumonia and if they were offered therapeutic trial. Those who failed to respond to the treatment were later included into the study if their sputum was negative for AFB and if they were positive for HIV. Patients who could produce sputum were asked to give another set of sputum, after brushing their teeth and rinsing their mouth under tap water, for staining for *Pneumocystis jirovecii* (PJ). All patients who were ‘sputum smear’-negative for AFB underwent acid-fast stain after sputum liquefaction with sodium hypochlorite and concentration with a centrifuge and culture for *M. tuberculosis*. Those who tested negative for PJ in sputum sample and patients who could not produce sputum underwent bronchoscopy for bronchoalveolar lavage and post-bronchoscopy sputum examination for AFB and culture for *M. tuberculosis.*

### Bronchoscopic technique

Both the oral and nasal pharynges were anesthetized with 5 ml of 2% lidocaine solution using a sprayer before bronchoscopy. Transnasal fiber-optic bronchoscopy was performed with Olympus BF A-3R flexible bronchoscope after administering a local intranasal lidocaine jelly. The scope was then wedged to a segmental or sub-segmental bronchus of the abnormal area as determined by the chest X-ray and a lavage was performed by instilling between 80 and 120 ml of sterile normal saline and immediately sucking it with a suction machine. Bronchoalveolar lavage was continued until at least 50 ml of the instilled saline was recovered. As most of the patients were in respiratory distress, the procedure had to be done as quickly as possible. The tracheo-bronchial tree was also visualized for the presence of purulent secretion and localized lesions such as Kaposi sarcoma. Patients were monitored for their oxygen saturation and most required administration of intranasal oxygen. Post bronchoscopy, patients were asked to cough and produce sputum. The BAL fluid and post-bronchoscopy sputum were sent for acid-fast stain and culture.

### Sputum and BAL acid-fast stain by concentration technique.

Initial smear-negative sputa, already pooled and kept at +4°C and bronchoalveolar lavage were transferred to a 15 ml screw-capped tube and mixed with an equal volume of sodium hypochlorite (5%). The tubes were incubated at room temperature for 15 min and shaken by hand at regular intervals. After addition of 8 ml of distilled water, the tubes were centrifuged at 3,000 rpm for 15 min. The supernatant of each tube was carefully discarded, the sediment was mixed with remaining fluid and direct smears were prepared by applying a drop of the resuspended sediment with a sterile pipette to a slide. The slides were dried in air, heat-fixed and stained by the Ziehl Nielsen technique.[7]

### BAL, pre- and post-bronchoscopy sputum for AFB and culture for *M. tuberculosis*

AFS using the Ziehl-Nielsen method was initially done on all BAL specimens and post-bronchoscopy sputum cultures and the results employed for the management of patients. Subsequently, mycobacterial culture on conventional Löwenstain-Jensen (LJ) egg medium and LJ medium containing pyruvate was performed for all pre- and post-bronchoscopy sputum and BAL fluid samples. Before culturing, the samples were digested and decontaminated from nonmycobacterial microorganisms by the sodium lauryl sulfate method.[8] The tubes were incubated at 37°C in 5% CO_2_ for 1 week, at 37°C in air for another 7 weeks and thereafter were checked once a week for mycobacterial growth. Growth of mycobacteria was confirmed by typical colony morphology and microscopy for AFB. In order to identify the LJ-grown isolates to species level, the nitrate reductase test, thiophene 2 carboxylic acid hydroxide (TCH) and the pyrazinamide test were performed.

### Chest X-rays

Chest X-rays were read by two radiologists unaware of the diagnosis. Whenever there was discordance in the reading, they were asked to reach a consensus diagnosis. Chest X-rays were read as ‘highly likely’ (typical), ‘likely’ or ‘less likely’ of the various pulmonary opportunistic infections including tuberculosis based on an already prepared diagnostic protocol format.

### Ethical consideration

The study was approved by the National Ethical Committee in Ethiopia and the Ethical Committee of the Karolinska Institute in Sweden. HIV testing and bronchoscopy were done after informed consent. Patients were referred for antiretroviral treatment, care and social support whenever applicable.

### Statistical method

After pre-coding of the variables, the raw data was entered into the computer using EPI-Info version 6.04D statistical package and exported to SPSS for windows version 11 statistical package for the purpose of analysis. Analysis was done using both the statistical packages. Frequencies, proportions and summary statistics were used to describe the study subjects in relation to relevant variables. For comparing the results of different test procedures, sensitivity, specificity and predictive values were calculated along with their 95% CI.

## Results

**Figure 1:**
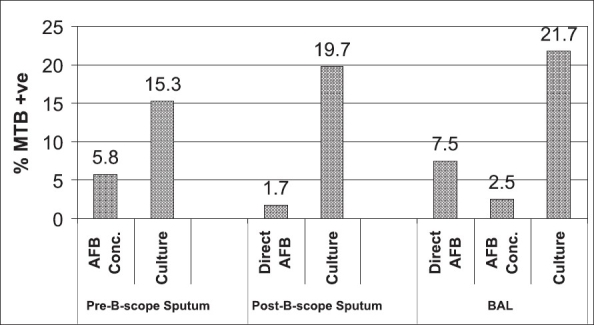
Prevalence of MTB in different speciment by different diagnostic techniques

A total of 131 patients were enrolled into the study, of whom 85 (64.9%) were able to produce sputum and underwent AFS after sputum liquefaction with sodium hypochlorite and concentration with centrifuge and culture for *mycobacterium tuberculosis*. Bronchoscopy and bronchoalveolar lavage were done in 120 patients after excluding 11 patients in whom sputum examination was positive for *P. jirovecii*. Post-bronchoscopy sputum was obtained in 117 of the 120 patients. The relative yield of the various diagnostic methods and comparison of the prevalence among the different techniques are depicted in Figure 1. A total of 27 (22.5%) patients were diagnosed to have tuberculosis, of whom 26 were from BAL culture and one from sputum culture. Pre-bronchoscopy sputum culture was positive in 13 (15.3%) of the 85 patients, of whom 5 (38.5%) could be diagnosed by sputum concentration technique. Nine patients were positive for AFB from BAL smear, of whom 3 were positive by sputum concentration too. Hence, a total of 11 (41%) patients out of the 27 culture-verified cases were smear positive either from sputum or BAL and immediate use of the results to initiate treatment was possible.

Post-bronchoscopy sputum smear for AFB had the lowest yield, with only two specimens positive for AFB; and both were positive in BAL smear. However, post-bronchoscopy sputum culture was as good as BAL culture (sensitivity 88.5%). The sensitivity, specificity, positive and negative predictive values of the various diagnostic tests against their gold standard are shown in Table 1.

**Table 1:** Comparison of various diagnostic tests against their gold standard

	Sputum concentrations V_s_ Sputum culture (%)	BAL AFB V_s_ BAL culture (%)	BAL concentrations V_s_ BAL culture (%)	Post B-scope Sputum AFB V_s_ culture (%)	Post B-scope sputum culture V_s_ BAL culture (%)
Sensitivity	5/13 (38.5)	9/26 (34.6)	3/26 (11.5)	2/23 (8.7)	23/26 (88.5)
Specificity	72/72 (100)	87/94 (92.6)	94/94 (100)	93/94 (98.9)	91/91 (100)
Positive predictive value	5/5 (100)	9/16 (56.3)	3/3 (100)	2/3 (66.7)	23/23 (100)
Negative predictive value	72/85 (90)	87/104 (83.7)	94/17 (80)	93/114 (81.6)	91/94 (96.8)

BAL - Bronchoalveolar lavage

Amongst the 85 patients who could produce sputum, all BAL, post-bronchoscopy and pre-bronchoscopy sputum cultures detected similar number of patients. BAL and post-bronchoscopy sputum cultures were positive in 12 (14%) patients each, while pre-bronchoscopy sputum culture was positive in 13 (15.3%) patients. BAL and post-bronchoscopy sputum, on the other hand, detected MTB in 14 (40%) and 11 (31.4%) of the 35 patients who could not produce sputum for this difference was statistically significant in the two groups for both BAL and post-bronchoscopy sputum (*P*= 0.002, *P*= 0.028 respectively). Besides, BAL smear was positive in only 3 (3.5%) cases amongst the 85 sputum producers, while it detected 6 (17%) amongst the sputum non-producers (*P*= 0.028).

Comparison of the radiological diagnosis to the culture-verified tuberculosis demonstrated that only 11(41%) patients were strongly suspected of having tuberculosis radiologically. The remaining patients were considered to have pneumocystis pneumonia and bacterial infection and were put on separate treatment until either the smear or culture results were available. The sensitivity and positive predictive value of chest X-ray in the diagnosis of tuberculosis amongst this group of patients were low.

## Discussion

The results of our study have demonstrated that in HIV-infected patients presenting with atypical chest X-ray and negative sputum smear, culture from bronchoalveolar lavage is the most sensitive method for detection of tuberculosis. The overall sensitivity of sputum culture was relatively low compared to BAL (15.3% versus 21.7%) and this is comparable to findings from other studies.[9–11] However, in patients who could produce sputum, the sensitivity of sputum culture was comparable to BAL and post-bronchoscopy sputum. Of note is the fact that 38.5% of the ‘sputum culture’-verified cases were positive in sputum smears after concentration with sodium hypochlorite. This is an important observation because the technique of sputum concentration is simple and affordable in most developing countries. Besides, it will avoid delay in the diagnosis and allow immediate initiation of treatment for tuberculosis. In HIV-positive patients in whom sputum is likely to be negative and the chest X-ray is likely to be misinterpreted as pneumocystis pneumonia or bacterial infection, the value of such method cannot be underestimated. Sputum concentration technique has been shown to improve detection of AFB in both HIV-infected and non-infected patients in the past.[6,7]

BAL smear was positive in one-third of the patients positive by BAL culture and was complementary to sputum smear by concentration technique. However, all the 6 patients that complemented sputum smear were from among patients who could not produce sputum. Results of both smear and culture suggest, to a significant level, that the major benefit of bronchoscopic aspirate is in patients who cannot produce sputum. The two quick methods of direct BAL smear and sputum AFS by concentration method allowed the detection of nearly half of the culture-proven cases. This is welcome news to the treating physician who, otherwise, had to wait 8 weeks before the diagnosis was made by sputum culture.

Post-bronchoscopy sputum culture is highly sensitive compared to BAL culture (sensitivity 88.5%). However, all cultures from post-bronchoscopy sputum were also positive in BAL culture and therefore did not add much to the overall diagnosis. It has been suggested in the past that the instillation of local anesthetic agents into the tracheo- bronchial tree during bronchoscopy will kill some of the mycobacteria and render the BAL less culture-positive compared to post-bronchoscopy sputum. Our finding, however, does not support this notion and BAL culture remains the most sensitive method for the diagnosis of tuberculosis.

Induced sputum has been shown to have an excellent yield compared to BAL and therefore can be a practical solution for those who cannot expectorate adequate sputum.[12–14] Unfortunately, this technique has not been evaluated under routine clinical conditions in developing countries. However, it is likely to be an inexpensive and a more practical method compared to bronchoscopy.

Several studies have demonstrated that transbronchial biopsy (TBBX) provides incremental diagnostic information not available from evaluation of sputum or bronchoalveolar lavage.[15–18] The procedure, however, is not without complications, particularly pneumothorax and this could mean a lot for an already seriously sick patient. Therefore, TBBX has to be done on selected patients who can tolerate the procedure and who preferably have a unilateral disease. We did not perform transbronchial biopsy in our patients.

As of recent, the polymerase chain reaction (PCR) has been shown to have a very high sensitivity and specificity in both sputum and BAL specimen and is increasingly being used for the diagnosis of tuberculosis.[19–21] The clinical usefulness of this technology, however, will be questionable for several reasons, including cost in resource-constrained countries like Ethiopia.

In conclusion, our study demonstrated that sputum AFS using the concentration method and BAL smear for AFB are rapid methods for the diagnosis of PTB and bronchoalveolar lavage is a biologic specimen with the highest yield and is the most useful. It has also demonstrated that the added value of BAL culture over sputum culture is in patients who cannot produce sputum. It is therefore reasonable to conclude, that in HIV-infected patients with negative sputum smear and atypical chest X ray, bronchoscopy as a diagnostic tool for PTB should be spared for patients who cannot produce sputum. Such a recommendation, however, requires a larger and more detailed study before it is implemented. We also recommend that sputum induction, being relatively simple and inexpensive, should be evaluated as an alternative for bronchoscopy in resource-constrained settings.
